# Global, regional, and national burden of brain and central nervous system cancer: a systematic analysis of incidence, deaths, and DALYS with predictions to 2040

**DOI:** 10.1097/JS9.0000000000002359

**Published:** 2025-04-01

**Authors:** Qi Zhang, Honghao Yu, Jue Zhong, Weiting Cheng, Yiwei Qi

**Affiliations:** a Department of Plastic and Cosmetic Surgery, Tongji Hospital, Tongji Medical College, Huazhong University of Science and Technology, Wuhan, China; b Department of Dermatology and Venereology, Peking University First Hospital, Beijing, China; c Department of Neurosurgery, Tongren Hospital of Wuhan University (Wuhan Third Hospital), Wuhan, P. R. China; d Department of Oncology, Cancer Center, Renmin Hospital of Wuhan University, Wuhan, China; e Department of Neurosurgery, Tongji Hospital, Tongji Medical College, Huazhong University of Science and Technology, Wuhan, China

**Keywords:** brain and CNS cancer, epidemiology, forecasting model, Global Burden of Disease

## Abstract

**Background:**

Brain and central nervous system (CNS) cancers present significant health challenges globally, characterized by increasing incidence and mortality rates. This study utilizes data from the Global Burden of Disease (GBD) 2021 to analyze trends and project future burdens.

**Methods:**

We calculated age-standardized rates (ASRs) of incidence, mortality, and disability-adjusted life years (DALYs) for brain and CNS cancers from 1990 to 2021. Trends were analyzed using estimated annual percentage change, and future projections were made with an Autoregressive Integrated Moving Average (ARIMA) model. Correlations between the socio-demographic index (SDI) and ASR were also examined.

**Results:**

The study revealed a 106% increase in incidence number and a 63.67% rise in death number over the study period. The ARIMA model predicts declines in incidence, mortality, and DALYs by 2040. Higher incidence rates were observed in high SDI regions, while greater mortality occurred in low SDI areas, indicating significant disparities.

**Conclusion:**

These findings underscore the need for targeted interventions and sustained healthcare investments to manage the global burden of brain and CNS cancers effectively. The projected declines suggest potential effectiveness of current public health strategies but highlight the importance of addressing socio-demographic disparities.

## Introduction

Brain and central nervous system (CNS) cancers represent some of the most challenging malignancies in oncology due to their profound impact on mortality, incidence, and the significant impairment they cause to the quality of life of patients^[^1^]^. These cancers are particularly aggressive and require complex, often highly individualized treatment strategies that can vary widely in their effectiveness^[^2^]^. The Global Burden of Disease (GBD) study serves as a cornerstone in global health assessment, providing a comprehensive view of disease burdens across different populations and regions^[^3^]^. By integrating vast datasets from multiple sources and applying sophisticated analytical frameworks, the GBD study reveals the distribution, trends, and consequences of health conditions^[^4,5^]^. Utilizing the extensive data from the GBD 2021 study, this research explores the changing dynamics of these malignancies from 1990 to 2021 and uses predictive modeling to estimate trends through to 2040. The primary aim of this study is to provide comprehensive insights that could guide the development of public health strategies, influence healthcare policies, and optimize resource allocation to improve the management and outcomes of brain and CNS cancers worldwide. Additionally, this research seeks to emphasize the critical need for global cooperation in enhancing diagnostic and treatment capabilities across diverse healthcare infrastructures.

**HIGHLIGHTS**

- Western Europe reported the highest incidence, and Central Europe exhibited the greatest mortality and disability-adjusted life year (DALY) burdens.
- Countries and territories with higher socio-demographic index tended to have higher age-standardized rates of incidence, mortality, and DALYs.
- The Autoregressive Integrated Moving Average model predicted a decline in incidence, mortality, and DALYs by 2040.

## Methods

Our methodology involved a rigorous extraction and sophisticated analysis of data related to the incidence, mortality, and disability-adjusted life years (DALYs) associated with brain and CNS cancers as recorded in the GBD 2021. This study spans a global scale, examining health data from 204 countries to provide a panoramic view of the disease burden. We utilized age-standardized rates (ASRs) and estimated annual percentage changes (EAPCs) to analyze trends over the past three decades comprehensively. For future trend predictions, we employed Autoregressive Integrated Moving Average (ARIMA) models, which are particularly adept at handling the complexities of time-series data typical of disease incidence and mortality statistics. We also examined the relationship between the disease burden and the socio-demographic index (SDI) to evaluate how socioeconomic factors influence the epidemiology of these cancers, providing a layered understanding of both historical and future disease patterns.

## Results

### Global trends of incidence, deaths, and DALYs

**Figure 1.**
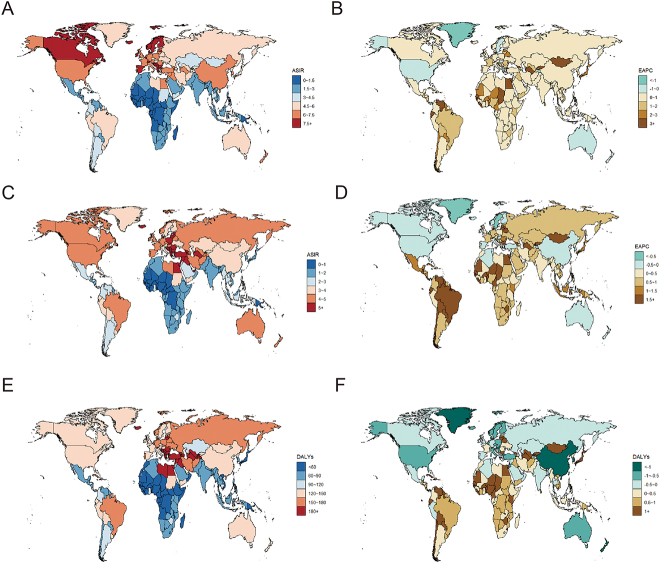
Global trends of incidence, deaths, and DALYs of brain and central nervous system cancer for both sexes in 204 countries and territories. (A) The age-standardized incidence rate (ASIR) in 2021; (B) the EAPC of ASIR; (C) age-standardized deaths rate (ASDR); (D) the EAPC of ASDR; (E) the age-standardized DALY rate; and (F) the EAPC of age-standardized DALY rate.

Our analysis uncovered a significant increase in the global incidence of brain and CNS cancers, with cases rising by 106% over the study period. This substantial increase likely reflects not only advancements in diagnostic technologies but also potential escalations in environmental and genetic risk factors (Fig. 1, Table 1). Mortality cases have escalated by 63.67%, underscoring the deadly nature of these cancers and the ongoing challenges in improving treatment outcomes (Fig. 1, Table 2). However, 10% reduction in ASR of DALYs indicates significant progress in disease management and patient care, which has enhanced the quality of life for many patients (Fig. 1, Table 3). Regionally, Western Europe displayed the highest incidence rates, suggesting superior diagnostic capabilities, whereas Central Europe showed the highest mortality and DALY rates, indicating variable treatment success and access to healthcare services.

**Table 1:** Global and regional trends of incidence.

Characteristic	Number in 1990 (95% CI)	ASR in 1990 (95% CI)	Number in 2021 (95% CI)	ASR in 2021 (95% CI)	EAPC (95% CI)
Global	173 086 (147 452, 194 951)	3.75 (3.21, 4.21)	357 482 (310 457, 407 433)	4.28 (3.71, 4.88)	0.44 (0.40 to 0.49)
High SDI	55 694 (54 173, 56 847)	5.66 (5.52, 5.78)	100 994 (94 964, 105 629)	6.38 (6.08, 6.64)	0.43 (0.36 to 0.50)
High-middle SDI	49 615 (42 242, 55 311)	4.81 (4.1, 5.35)	97 818 (83 423, 112 505)	5.9 (5.05, 6.77)	0.68 (0.63 to 0.74)
Middle SDI	47 058 (35 595, 56 517)	3.35 (2.57, 4.07)	106 918 (86 956, 128 833)	4.11 (3.34, 4.94)	0.67 (0.65 to 0.70)
Low-middle SDI	15 847 (11 648, 21 806)	1.73 (1.31, 2.32)	39 604 (31 858, 49 414)	2.34 (1.89, 2.93)	1.04 (0.98 to 1.09)
Low SDI	4669 (2949, 7298)	1.23 (0.77, 1.75)	11 810 (8190, 15 144)	1.43 (1, 1.82)	0.46 (0.36 to 0.55)
East Asia	48 578 (35 372, 60 360)	4.63 (3.39, 5.77)	107 614 (83 225, 135 768)	6.02 (4.69, 7.53)	0.82 (0.78 to 0.86)
Southeast Asia	7051 (5029, 8699)	1.97 (1.43, 2.45)	16 691 (12 170, 19 950)	2.4 (1.76, 2.87)	0.67 (0.56 to 0.77)
Oceania	32 (16, 43)	0.69 (0.35, 0.94)	82 (43, 110)	0.74 (0.38, 0.99)	0.26 (0.19 to 0.32)
Central Asia	1966 (1640, 2244)	3.24 (2.68, 3.69)	4636 (4013, 5300)	4.94 (4.29, 5.62)	1.54 (1.40 to 1.69)
Central Europe	8003 (7657, 8532)	5.76 (5.5, 6.15)	11 721 (10 689, 12 787)	6.64 (6.07, 7.25)	0.55 (0.30 to 0.80)
Eastern Europe	9101 (8400, 9678)	3.63 (3.37, 3.85)	14 293 (13 134, 15 494)	4.98 (4.6, 5.38)	0.95 (0.85 to 1.04)
High-income Asia Pacific	5649 (5037, 5978)	3.11 (2.76, 3.29)	16 368 (14 108, 18 001)	5.44 (4.76, 5.92)	2.02 (1.71 to 2.33)
Australasia	1393 (1343, 1448)	6.32 (6.1, 6.56)	2536 (2342, 2730)	5.95 (5.54, 6.38)	−0.14 (-0.23 to -0.06)
Western Europe	30 521 (29 740, 31 118)	6.55 (6.4, 6.66)	49 619 (46 606, 51 779)	7.44 (7.13, 7.71)	0.48 (0.36 to 0.61)
Southern Latin America	1485 (1350, 1622)	3.12 (2.84, 3.41)	2981 (2776, 3173)	3.81 (3.55, 4.06)	1.13 (0.83 to 1.43)
High-income North America	22 170 (21 558, 22 637)	7.11 (6.93, 7.24)	36 462 (34 376, 37 686)	7.08 (6.75, 7.31)	0.02 (-0.05 to 0.09)
Caribbean	897 (824, 1131)	2.93 (2.72, 3.61)	1971 (1725, 2270)	3.85 (3.37, 4.46)	1.25 (1.13 to 1.37)
Andean Latin America	838 (665, 1161)	2.76 (2.2, 3.73)	2794 (2222, 3479)	4.43 (3.52, 5.5)	1.70 (1.39 to 2.02)
Central Latin America	2825 (2740, 2916)	2.23 (2.17, 2.29)	7586 (6775, 8533)	2.98 (2.66, 3.36)	0.71 (0.44 to 0.99)
Tropical Latin America	4967 (4716, 5251)	4.14 (3.93, 4.4)	14 101 (13 434, 14 673)	5.65 (5.37, 5.88)	1.09 (0.85 to 1.33)
North Africa and Middle East	10 151 (7656, 14 315)	4.09 (3.16, 5.72)	26 566 (19 734, 32 479)	4.98 (3.7, 6.1)	0.85 (0.75 to 0.94)
South Asia	13 985 (9078, 18 699)	1.61 (1.05, 2.12)	31 817 (26 009, 43 157)	1.87 (1.53, 2.55)	0.34 (0.23 to 0.44)
Central Sub-Saharan Africa	295 (213, 419)	0.83 (0.6, 1.07)	876 (586, 1182)	1.03 (0.67, 1.39)	0.80 (0.67 to 0.93)
Eastern Sub-Saharan Africa	1848 (1234, 2725)	1.25 (0.81, 1.67)	4821 (3457, 6278)	1.53 (1.05, 1.92)	0.70 (0.64 to 0.75)
Southern Sub-Saharan Africa	569 (441, 729)	1.57 (1.2, 1.98)	1390 (1024, 1668)	2.05 (1.5, 2.43)	0.91 (0.82 to 1.01)
Western Sub-Saharan Africa	762 (515, 997)	0.43 (0.3, 0.53)	2558 (1315, 3276)	0.63 (0.34, 0.79)	1.47 (1.38 to 1.57)

CI, confidence interval.

**Table 2:** Global and regional trends of deaths.

Characteristic	Number in 1990 (95% CI)	ASR in 1990 (95% CI)	Number in 2021 (95% CI)	ASR in 2021 (95% CI)	EAPC (95% CI)
Global	136 219 (114 799, 155 197)	3.04 (2.58, 3.45)	258 627 (222 185, 296 134)	3.06 (2.62, 3.5)	0.18 (0.12 to 0.24)
High SDI	37 798 (36 603, 38 634)	3.69 (3.57, 3.76)	63 268 (59 449, 66 058)	3.54 (3.36, 3.68)	0.05 (-0.01 to 0.12)
High-middle SDI	40 529 (34 407, 45 442)	3.96 (3.36, 4.44)	71 070 (60 266, 81 226)	3.94 (3.36, 4.51)	0.12 (0.05 to 0.18)
Middle SDI	39 418 (30 141, 47 589)	2.97 (2.31, 3.63)	79 779 (64 859, 96 617)	3.02 (2.45, 3.65)	0.24 (0.17 to 0.32)
Low-middle SDI	14 061 (10 404, 19 412)	1.62 (1.23, 2.18)	33 996 (27 308, 42 717)	2.08 (1.68, 2.62)	0.94 (0.89 to 0.99)
Low SDI	4240 (2670, 6641)	1.18 (0.74, 1.68)	10 225 (7080, 13 086)	1.33 (0.93, 1.69)	0.32 (0.28 to 0.37)
East Asia	40 077 (29 283, 50 223)	3.99 (2.94, 4.96)	70 565 (53 460, 90 082)	3.59 (2.72, 4.53)	−0.26 (-0.34 to -0.18)
Southeast Asia	6081 (4373, 7517)	1.82 (1.33, 2.27)	14 216 (10 394, 17 094)	2.08 (1.54, 2.5)	0.71 (0.61 to 0.81)
Oceania	28 (14, 38)	0.65 (0.34, 0.88)	71 (37, 96)	0.69 (0.36, 0.92)	0.16 (0.12 to 0.20)
Central Asia	1725 (1431, 1967)	2.94 (2.42, 3.35)	3999 (3464, 4566)	4.34 (3.77, 4.92)	1.36 (1.26 to 1.46)
Central Europe	7157 (6848, 7639)	5.05 (4.83, 5.4)	10 772 (9820, 11 748)	5.57 (5.09, 6.08)	0.64 (0.50 to 0.79)
Eastern Europe	7941 (7360, 8419)	3.11 (2.9, 3.29)	12 717 (11 709, 13 758)	4.17 (3.86, 4.5)	0.99 (0.92 to 1.07)
High-income Asia Pacific	2332 (1967, 2479)	1.25 (1.05, 1.33)	4906 (4245, 5348)	1.51 (1.31, 1.63)	0.59 (0.44 to 0.75)
Australasia	1096 (1060, 1132)	4.85 (4.69, 5.01)	1959 (1799, 2121)	4.21 (3.9, 4.52)	−0.31 (-0.39 to -0.24)
Western Europe	21 670 (21 089, 22 079)	4.33 (4.24, 4.41)	33 436 (31 382, 34 905)	4.34 (4.14, 4.5)	0.20 (0.14 to 0.27)
Southern Latin America	1243 (1130, 1356)	2.63 (2.39, 2.87)	2306 (2149, 2456)	2.83 (2.63, 3.02)	0.68 (0.50 to 0.87)
High-income North America	14 578 (14 099, 14 871)	4.46 (4.33, 4.54)	23 967 (22 466, 24 781)	4.09 (3.87, 4.22)	−0.13 (-0.19 to -0.06)
Caribbean	728 (666, 936)	2.46 (2.26, 3.05)	1634 (1424, 1901)	3.16 (2.76, 3.68)	0.99 (0.89 to 1.08)
Andean Latin America	721 (573, 986)	2.51 (2, 3.36)	2199 (1751, 2725)	3.55 (2.83, 4.4)	1.25 (1.06 to 1.44)
Central Latin America	2332 (2267, 2403)	1.97 (1.92, 2.03)	6167 (5505, 6892)	2.44 (2.17, 2.72)	1.27 (0.97 to 1.56)
Tropical Latin America	4216 (4007, 4469)	3.69 (3.51, 3.94)	12 102 (11 477, 12 611)	4.8 (4.55, 5.01)	1.65 (1.39 to 1.90)
North Africa and Middle East	8735 (6654, 12 267)	3.91 (3.03, 5.52)	22 280 (16 570, 27 228)	4.44 (3.31, 5.43)	0.52 (0.45 to 0.59)
South Asia	12 481 (8119, 16 644)	1.51 (0.98, 1.98)	27 080 (22 307, 37 097)	1.63 (1.35, 2.24)	0.33 (0.25 to 0.41)
Central Sub-Saharan Africa	272 (199, 381)	0.81 (0.58, 1.05)	786 (526, 1071)	0.99 (0.65, 1.35)	0.61 (0.53 to 0.68)
Eastern Sub-Saharan Africa	1653 (1096, 2427)	1.19 (0.76, 1.57)	4121 (2903, 5341)	1.41 (0.94, 1.77)	0.52 (0.49 to 0.55)
Southern Sub-Saharan Africa	498 (383, 634)	1.46 (1.1, 1.83)	1231 (901, 1462)	1.88 (1.36, 2.22)	0.82 (0.74 to 0.90)
Western Sub-Saharan Africa	657 (451, 869)	0.39 (0.28, 0.49)	2113 (1104, 2719)	0.56 (0.31, 0.7)	1.21 (1.14 to 1.28)

CI, confidence interval.

**Table 3:** Global and regional trends of DALYs.

Characteristic	Number in 1990 (95% CI)	ASR in 1990 (95% CI)	Number in 2021 (95% CI)	ASR in 2021 (95% CI)	EAPC (95% CI)
Global	5 958 481 (4 871 422, 6 901 955)	119.88 (99.23, 137.57)	8 912 595 (7 612 511, 10 356 061)	107.91 (91.74, 125.59)	−0.38 (-0.42 to -0.34)
High SDI	1 312 840 (1 274 153, 1 335 629)	138.42 (134.16, 140.88)	1 781 113 (1 708 716, 1 852 581)	121.28 (117, 126.53)	−0.41 (-0.47 to -0.35)
High-middle SDI	1 714 312 (1 420 346, 1 935 787)	165.43 (136.86, 186.78)	2 227 122 (1 905 892, 2 570 920)	139.39 (119.25, 161.1)	−0.67 (-0.75 to -0.60)
Middle SDI	1 947 466 (1 457 387, 2 303 974)	123.37 (93.26, 147.67)	2 877 443 (2 335 479, 3 497 679)	111.02 (89.88, 134.25)	−0.40 (-0.45 to -0.36)
Low-middle SDI	739 174 (539 762, 1 025 047)	68.09 (50.04, 94.17)	1 473 738 (1 172 367, 1 835 598)	81.83 (65.23, 102.18)	0.66 (0.60 to 0.73)
Low SDI	237 683 (149 533, 396 650)	49.14 (30.84, 76.19)	543 981 (373 054, 698 812)	54.23 (37.56, 69.66)	0.30 (0.22 to 0.38)
East Asia	1 925 699 (1 364 552, 2 345 360)	171.93 (122.56, 210.22)	2 304 510 (1 764 775, 2 942 852)	132.93 (102.06, 169.28)	−1.07 (-1.17 to -0.96)
Southeast Asia	292 573 (204 725, 369 849)	71.53 (51.17, 88.97)	555 834 (402 955, 665 412)	78.49 (57.05, 93.86)	0.30 (0.19 to 0.41)
Oceania	1465 (718, 2014)	25.7 (12.67, 34.76)	3607 (1910, 4831)	28.13 (14.76, 37.7)	0.30 (0.26 to 0.35)
Central Asia	86 683 (73 174, 100 754)	131.46 (110.1, 151.07)	176 070 (153 099, 202 565)	182.25 (158.84, 209.36)	1.20 (1.05 to 1.35)
Central Europe	278 998 (266 528, 297 734)	208.8 (199.43, 223.39)	303 122 (276 834, 330 259)	186.47 (169.76, 203.97)	−0.29 (-0.46 to -0.11)
Eastern Europe	341 280 (319 142, 359 419)	143.55 (135.1, 150.63)	412 932 (381 399, 448 020)	156.28 (144.98, 168.52)	0.11 (0.00 to 0.22)
High-income Asia Pacific	99 317 (83 223, 105 743)	57.29 (47.96, 61.29)	143 487 (124 838, 154 975)	62.99 (54.86, 67.63)	0.29 (0.02 to 0.57)
Australasia	37 275 (36 194, 38 458)	173.89 (168.88, 179.23)	56 637 (52 908, 60 433)	142.13 (133.15, 151.17)	−0.68 (-0.78 to -0.58)
Western Europe	721 095 (708 900, 731 626)	164.09 (161.79, 166.3)	905 401 (868 843, 936 529)	146.61 (142.42, 151.33)	−0.32 (-0.39 to -0.26)
Southern Latin America	46 204 (41 943, 50 383)	95.43 (86.64, 104.16)	74 320 (69 348, 79 252)	100.03 (93.34, 106.54)	0.62 (0.32 to 0.92)
High-income North America	480 948 (471 488, 488 300)	158.96 (156.25, 161.21)	665 532 (637 599, 683 259)	134.63 (130.28, 138.3)	−0.50 (-0.58 to -0.42)
Caribbean	32 978 (29 027, 46 110)	100.78 (90.24, 136.18)	59 372 (50 989, 71 992)	120.4 (102.12, 149.12)	0.91 (0.81 to 1.01)
Andean Latin America	38 022 (30 031, 53 410)	106.44 (85.4, 147.72)	88 267 (70 227, 109 896)	137.13 (109.15, 170.75)	0.98 (0.65 to 1.30)
Central Latin America	121 506 (117 569, 126 440)	81.28 (79.06, 83.83)	231 645 (206 113, 261 520)	90.87 (80.7, 102.84)	0.22 (-0.02 to 0.46)
Tropical Latin America	195 773 (184 388, 206 372)	145.58 (137.88, 153.4)	412 632 (394 769, 428 785)	168.28 (160.46, 175.42)	0.58 (0.32 to 0.85)
North Africa and Middle East	422 026 (315 099, 603 641)	146.05 (111.15, 206.33)	879 767 (652 323, 1 063 848)	153.92 (114.39, 185.97)	0.34 (0.26 to 0.43)
South Asia	656 447 (417 585, 895 866)	64.14 (41.51, 86.08)	1 183 610 (959 567, 1 598 986)	67.11 (54.35, 90.91)	0.03 (-0.06 to 0.12)
Central Sub-Saharan Africa	14 014 (9724, 21 832)	30.52 (22.42, 41.39)	37 965 (25 872, 50 473)	35.97 (23.92, 49.18)	0.67 (0.54 to 0.79)
Eastern Sub-Saharan Africa	100 938 (67 629, 157 895)	51.55 (34.2, 73.61)	237 288 (172 511, 314 193)	60.09 (42, 77.85)	0.59 (0.52 to 0.65)
Southern Sub-Saharan Africa	22 631 (17 664, 29 444)	53.42 (41.19, 69.03)	50 627 (37 684, 61 397)	69.14 (51.2, 83.14)	0.89 (0.76 to 1.02)
Western Sub-Saharan Africa	42 610 (28 163, 58 275)	19.27 (13.32, 24.95)	129 970 (66 337, 169 012)	26.09 (13.68, 33.62)	1.21 (1.11 to 1.32)

CI, confidence interval.

### Correlation with SDI

**Figure 2.**
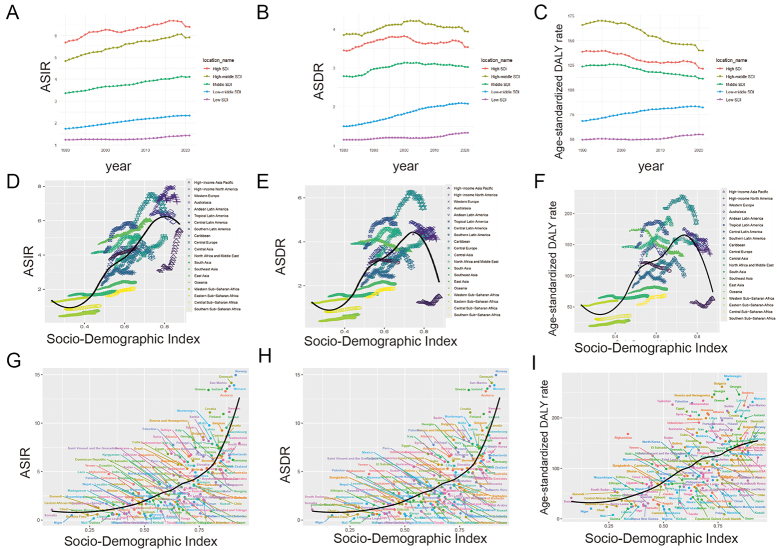
Age-standardized rates of incidence, death, and DALY of brain and central nervous system cancer and SDI from 1990 to 2021, by GBD regions and country and territory. (A) Age-standardized incidence rate (ASIR) in five SDI regions. (B) Age-standardized deaths (ASDR) in five SDI regions. (C) Age-standardized DALY rate in five SDI regions. (D) Age-standardized DALY rate (E) ASDR in GDB regions by SDI. (F) Age-standardized DALY rate. (G) ASIR in different countries and territories by SDI. (H) ASDR in different countries and territories by SDI. (I) Age-standardized DALY rate in different countries and territories by SDI.

The data indicated a strong positive correlation between higher SDI values and increased incidence rates, suggesting that more developed regions have better disease detection and reporting systems. However, the inverse relationship observed with mortality rates in lower SDI regions highlights the critical deficiencies in treatment and healthcare infrastructure, which are essential for improving patient survival. This disparity underscores the urgent need for targeted health interventions and policy initiatives that aim to equalize healthcare access and quality across different regions (Fig. 2).

### Prediction of brain and CNS cancer ASR to 2040

**Figure 3.**
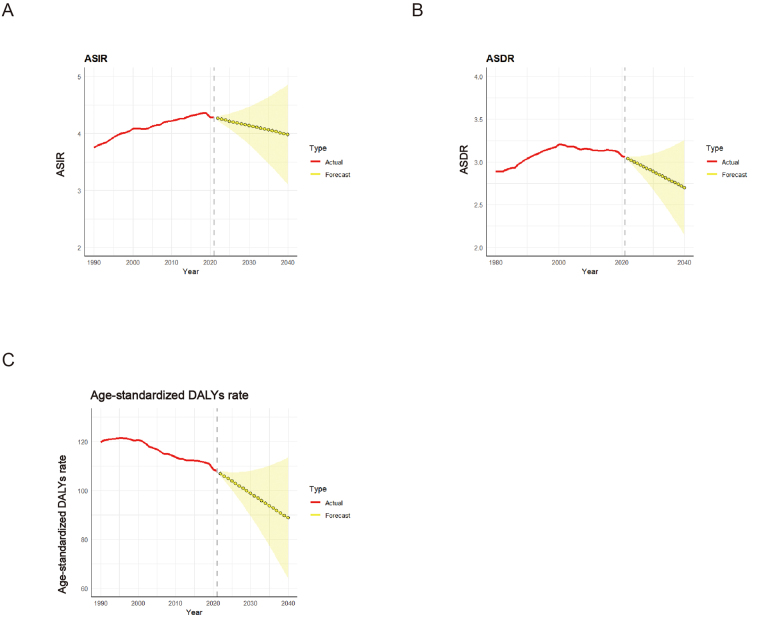
Predicted trends of global age-standardized rates of brain and central nervous system cancer in the next 19 years (2022–2040). (A) Predicted trends of age-standardized incidence rate. (B) Predicted trends of age-standardized deaths rate. (C) Predicted trends of age-standardized DALY rate.

The ARIMA model was used to quantitatively depict the trends of brain and CNS cancer incidence, mortality, and DALYs to 2040. The incidence was expected to decrease from 4.28 in 2021 to 3.98 per 100 000 in 2040 (Fig. 3A). The predicted mortality rate also kept decreasing from 3.06 in 2021 to 2.70 per 100 000 in 2040 (Fig. 3B). Further predictions of age-standardized DALY rate also decreased from 108 to 89 per 100 000 people (Fig. 3C).

## Discussion

From 1990 to 2021, the observed increase in incidence and mortality reflects the growing impact of brain and CNS cancers on global health. This rise can be attributed to various factors, including an aging population, improved diagnostic capabilities leading to better detection rates, changes in lifestyle and environmental risk factors, and advancements in cancer treatments that extend survival rates^[^6^]^. Despite medical progress, mortality rates have remained stable, highlighting ongoing challenges in early detection and treatment efficacy^[^7^]^. While the reduction in DALYs indicates some success in managing disease burden, the high absolute number of deaths highlights the need for improved treatment strategies. The necessity of further investment in research and the development of new therapies is crucial, alongside enhancing existing treatments and strengthening palliative care to improve patient quality of life^[^8^]^. The stark disparities observed across different SDI regions emphasize the essential need for improved healthcare policies that address the unique challenges faced by lower SDI regions. The ARIMA model predicts a declining trend in the incidence, mortality, and DALYs related to brain and CNS cancers by 2040. This outlook suggests that ongoing cancer prevention, early detection programs, and innovative treatments may lead to positive outcomes in the coming years. The anticipated reduction in disease burden highlights the potential impact of current public health initiatives and shows the importance of continued investment in research and healthcare infrastructure to sustain these positive trends^[^9^]^. Ongoing attention and efforts are crucial for ensuring the continued decline of these trends and improving patient quality of life. These policies should prioritize the enhancement of healthcare infrastructure, the expansion of access to advanced diagnostic tools, and the improvement of treatment modalities to effectively reduce mortality rates and enhance overall health outcomes.

## Conclusion

This study provides a comprehensive overview of global trends and future projections for brain and CNS cancers, emphasizing the need for sustained healthcare improvements and targeted policy interventions to effectively manage and mitigate the burden of these diseases globally. The projections offer hope but also underscore the necessity for ongoing efforts to ensure these positive outcomes are achieved.

## Data Availability

All the datasets displayed in this study can be obtained in the article. Further questions can be directed to the corresponding author.
